# Elevated Plasma Soluble CD14 and Skewed CD16+ Monocyte Distribution Persist despite Normalisation of Soluble CD163 and CXCL10 by Effective HIV Therapy: A Changing Paradigm for Routine HIV Laboratory Monitoring?

**DOI:** 10.1371/journal.pone.0115226

**Published:** 2014-12-29

**Authors:** Alison Castley, Cassandra Berry, Martyn French, Sonia Fernandez, Romano Krueger, David Nolan

**Affiliations:** 1 Molecular and Biomedical Sciences, School of Veterinary and Life Sciences, Murdoch University, Murdoch, Perth, Western Australia, Australia; 2 Institute for Immunology and Infectious Diseases, Murdoch University, Murdoch, Perth, Western Australia, Australia; 3 Department of Clinical Immunology, Royal Perth Hospital, Wellington Street, Perth, Western Australia, Australia; 4 School of Pathology and Laboratory Medicine, University of Western Australia, Nedlands, Perth, Western Australia, Australia; COCHIN INSTITUTE, Institut National de la Santé et de la Recherche Médicale, France

## Abstract

**Objective:**

We investigated plasma and flow cytometric biomarkers of monocyte status that have been associated with prognostic utility in HIV infection and other chronic inflammatory diseases, comparing 81 HIV^+^ individuals with a range of treatment outcomes to a group of 21 healthy control blood donors. Our aim is to develop and optimise monocyte assays that combine biological relevance, clinical utility, and ease of adoption into routine HIV laboratory practice.

**Design:**

Cross-sectional evaluation of concurrent plasma and whole blood samples.

**Methods:**

A flow cytometry protocol was developed comprising single-tube CD45, CD14, CD16, CD64, CD163, CD143 analysis with appropriately matched isotype controls. Plasma levels of soluble CD14 (sCD14), soluble CD163 (sCD163) and CXCL10 were measured by ELISA.

**Results:**

HIV status was associated with significantly increased expression of CD64, CD143 and CD163 on CD16^+^ monocytes, irrespective of the virological response to HIV therapy. Plasma levels of sCD14, sCD163 and CXCL10 were also significantly elevated in association with viremic HIV infection. Plasma sCD163 and CXCL10 levels were restored to healthy control levels by effective antiretroviral therapy while sCD14 levels remained elevated despite virological suppression (p<0.001).

**Conclusions:**

Flow cytometric and plasma biomarkers of monocyte activation indicate an ongoing systemic inflammatory response to HIV infection, characterised by persistent alterations of CD16^+^ monocyte expression profiles and elevated sCD14 levels, that are not corrected by antiretroviral therapy and likely to be prognostically significant. In contrast, sCD163 and CXCL10 levels declined on antiretroviral therapy, suggesting multiple activation pathways revealed by these biomarkers. Incorporation of these assays into routine clinical care is feasible and warrants further consideration, particularly in light of emerging therapeutic strategies that specifically target innate immune activation in HIV infection.

## Introduction

Monocytes are a heterogeneous cell population arising from the myeloid lineage that provide a link between innate and adaptive immunity. They can be classified according to cell surface expression of CD14 (a lipopolysaccharide receptor) and CD16 (FcγRIII, a low affinity Fc receptor) into three subsets known to have different phenotype and functions. The more numerous “classical” CD14^++^/CD16^-^ monocytes appear to be more granulocyte-like in that they are well-equipped for innate immune responses involving trans-endothelial migration and phagocytosis [1], while the remaining CD16^+^ monocytes have been more recently subclassified into “intermediate” (CD14^++^/CD16^+^) and “non-classical” (CD14^+^/CD16^++^) populations that appear to have more in common with dendritic cells and macrophages [1], in that they exhibit greater potential for HLA-restricted antigen presentation and pro-inflammatory cytokine production [2], migrating in response to distinct subset-specific chemokine/ligand gradients [3]. With regard to HIV infection, there has been a great deal of interest in the potential role of CD16^+^ monocytes, particularly CD14^++^/CD16^+^ intermediate monocytes, in disease pathogenesis given that these populations (which co-express CCR5) [3] are permissive to HIV infection [4] and are capable of transferring HIV infection across the genital mucosal barrier [5] as well as into the central nervous system [6]. Expansion of this intermediate monocyte population also appears to be associated with cardiovascular events in subjects referred for elective coronary angiography [7] and has been noted in acute coronary syndromes as well as in chronic HIV infection [8].

Additional cell surface markers may further characterise CD16^+^ monocyte function, particularly in the context of HIV infection and/or inflammation. These include the angiotensin converting enzyme (CD143), expressed predominantly on CD14^++^/CD16^+^ intermediate monocytes, which has been linked to mortality and cardiovascular disease in haemodialysis patients [9]; as well as the scavenger receptor CD163 which has been shown to be significantly elevated in the context of HIV infection [10], [11]. In contrast, the high affinity Fcγ receptor 1 (CD64) is principally expressed on CD14^++^/CD16^-^ classical monocytes, where it may serve as a biomarker of type I interferon activation in autoimmune diseases [12]. Monocyte CD64 expression may restrict productive HIV-1 infection by facilitating viral phagocytosis and degradation [13].

Several plasma biomarkers of monocyte activity have also been linked to HIV disease progression, including soluble CD14 (sCD14) which has been shown to predict all-cause mortality in HIV patients [14], even in the setting of undetectable plasma HIV RNA levels that would generally define effective HIV therapy [15], [16]. Elevated levels of soluble CD163 (sCD163) have also been shown to be associated with arterial inflammation and cardiovascular disease in HIV-infected patients [16], although in this case sCD163 levels appear responsive to HIV therapy [17]. Similarly, plasma levels of CXCL10 (also known as interferon gamma-induced protein 10 [IP-10]) are induced by HIV infection [18] but are also readily reduced by effective therapy [19].

Taken together, these data suggest that laboratory evaluation of monocyte populations may provide important prognostic information during HIV management that is currently ‘invisible’ through standard assessments of plasma HIV RNA levels and CD4 T cell counts. In this study we have therefore sought to develop and optimise methods that reveal monocyte status in a manner that combines biological relevance, clinical utility, and ease of adoption into routine HIV laboratory practice.

## Methods

Patients attending the Royal Perth Hospital (RPH) Immunology clinic and healthy control blood donors attending the Australian Red Cross Blood Service (ARCBS) were recruited for this study. Informed written consent was obtained from the patients participating in this investigation. This consent form was reviewed and accepted by the ethics committees.Written ethics committee approvals for this investigation were also received from Royal Perth Hospital (EC2012/170), Murdoch University (2012/216) and the Australian Red Cross Blood Service (11-08WA-15). After site approvals and patient consent, whole blood was collected into EDTA anti-coagulated tubes from 81 HIV-1 positive patients and 21 healthy controls.

### Sample Collection

Plasma was collected from EDTA whole blood samples within 6 hours of collection by centrifugal force of 2000 rpm for 20 mins. Plasma was removed and stored at −80°C until required for the enzyme-linked immunosorbent assay (ELISA). For flow cytometry analysis, whole blood (WB) samples were kept at room temperature and processed within 24 hours after collection.

### Whole blood flow cytometric analysis

The eight monoclonal antibodies (mAbs) utilised in this study were designed using the BD FACSelect multicolour panel designer then purchased from BD Biosciences, unless otherwise indicated, and stored at 4°C until required.

After assessing the effect of storage time on whole blood samples, mAb titrations and the effect of washing cell preparations (data not shown) we assessed the above cohorts using an 8 channel multicolour flow cytometer (FACSCanto II) for measuring monocytes and monocyte activation status using the following conjugated antibodies; 0.5 µl APC-H7 mouse anti-human CD14 (clone MφP9), 1 µl V450 mouse anti-human CD16 (clone 3G8), 1 µl V500 mouse anti-human CD45 (clone HI30), 1 µl PerCP-Cy5.5 mouse anti-human CD56 (clone B159), 2.5 µl PE-Cy7 mouse anti-human CD64 (clone 10.1-this was sub-aliquoted and stored to prevent degradation from repeated exposure to light) and 5 µl APC mouse anti-human hematopoietic progenitor cell (clone BB9), 1 µl anti-human CD163-Alexa Fluor488 (clone 215927) and 2 µl anti-human NKG2C-phycoerythrin (clone 134591) from R&D systems. The appropriately matched isotype controls were utilised for each mAb, as negative controls, and fluorescence minus one (FMO) staining was also utilised as a control procedure.

The mAbs were pipetted into a 3DT tube (BD Falcon) followed by the addition of 50 µl of well-mixed, reverse pipette WB. After gentle mixing the sample tubes were incubated for 15 min at room temperature in the dark. Red blood cells were lysed by adding 500 µl of 1× FACSLyse red cell lysis buffer (BD Biosciences), gently mixed then incubated for a further 15 min at room temperature in the dark. The stained cell preparation was acquired on the flow cytometer within 30 mins after incubation.

Sample acquisition was performed using 3-laser, 8-color configuration on the FACSCanto II flow cytometer with FACSDiva 6.1.1 software (BD Biosciences). The performance and quality control of the instrument was assessed and recorded daily utilising CST beads (BD Biosciences).

Fluorescence compensation was required and assessed as per kit instructions with anti-mouse Igκ CompBeads (BD Biosciences) as well as the above mentioned mAbs. Compensation values were calculated automatically and accepted when all fluorochrome-paired values were <30%. Voltages for forward scatter (FSC) and side scatter (SSC) were adjusted giving a FSC voltage of 385 and a SSC voltage of 450. This enabled distinct and clear separation of the monocyte cell population from lymphocyte and granulocyte cell populations. Sample preparation tubes were acquired by collecting 100,000 events using the acquisition gate on the lymphocyte cell population, ensuring at least 1000–5000 events were collected for the monocyte population.

### Analysis of monocyte subsets and activation markers

After successful acquisition the data files were saved to the BD FACS database then exported. Data was imported into Kaluza Flow Cytometric analysis software (version 1.1) where monocyte analysis was performed.

Duplets were removed from the analysis by gating on the singleton cells from a FSC-Area and FSC-Height plot. Lymphocytes and monocytes were visualised and gated on by using forward scatter/side scatter (FSC/SSC) dot plot. Monocyte subsets were identified by plotting CD45/FSC-A from the monocyte gate, then plotting the CD14/CD16 fluorescence from the CD45^+^ gate. The three monocyte subsets were defined by using isotype controls and FMO as cutoffs (classical monocytes (CD14^++^/CD16^−^), the intermediate monocytes (CD14^++^/CD16^+^) and the non-classical monocytes (CD14^+^/CD16^++^). To determine the presence of monocyte activation markers, CD64, CD163 or CD143 were plotted against SSC-A from each monocyte subset (i.e. classical, intermediate and non classical). The percentage of cells positive was determined from the number present in the parent group.

### Measurement of plasma sCD14, sCD163 and CXCL10 levels by ELISA

For the quantitative determination of plasma biomarker levels, ELISAs were utilised as per manufacturer's recommendations unless otherwise stated. R&D Systems, Quantikine ELISAs were used to determine sCD14, sCD163 and CXCL10 levels. Each sample was assayed in duplicate then the optical densities (OD) were measured at an absorbance of 450 nm and 570 nm. The latter wavelength corrects for any optical imperfections and is therefore subtracted from the 450 nm. Standards and controls were included as per kit instructions. Log/log standard curves were generated by averaging optical density readings from the standard results. To determine biomarker concentrations, plasma sample OD readings were also averaged and concentrations were determined from the log/log standard curve.

### Statistical analysis

Statistical data analysis was performed using SPSS version 21. Normality of data distribution was assessed using the Shapiro-Wilk test. Based on these results, one way ANOVA and Mann-Whitney tests were utilised to compare HIV+ and healthy control groups, with appropriate correction for multiple comparisons when >2 groups were compared. Bivariate correlation and multivariate linear regression analyses (with logarithmic data transformation to normalise distribution where appropriate) were utilised to estimate associations between plasma biomarkers and monocyte subsets with HIV status, HIV viral load, absolute CD4 counts, CD4:8 ratio, gender and age.

## Results

### Influence of HIV status on monocyte populations by flow cytometry analysis

The study population included 81 individuals with HIV infection and 21 healthy controls, covering a wide range of CD4 T cell counts and plasma HIV RNA levels and an age range from 20-70 years (Table 1). Within the HIV group there were 48 participants with viral load results <40 copies/mL (31 male, 17 female), 12 with viral load levels between 40 and 400 copies/mL (11 male, 1 female), and 21 with viral load levels >400 copies/mL (15 male, 6 female).

**Table 1:** Demographic and clinical characteristics of HIV-infected and healthy control subjects.

Category	Subcategory	HIV^+^ group	Controls
Gender	Males (n)	57	10
	Females (n)	24	11
	Total (n)	81	21
Age (years)	Males mean (range)	45 (20–65)	56 (45–70)
	Females mean (range)	37 (25–59)	41 (23–66)
Percentage CD4 (%)	Males mean (range)	27 (5–53)	NT
	Females mean (range)	28 (4–48)	NT
Absolute CD4 (cells/µL)	Males mean (SD, range)	603 (301, 140–1369)	NT
	Females mean (SD, range)	611 (324, 104–1188)	NT
CD4:CD8 ratio	Males mean (SD, range)	0.63 (0.41, 0.07–2.36)	NT
	Females mean (SD, range)	0.71 (0.38, 0.07–1.48)	NT
HIV viral load (copies/ml)	Males mean (SD, range)	30,905 (84,837, <40–446684)	NT
	Females mean (SD, range)	16,662 (43240, <40–173780)	NT
Duration of HIV (years)	Males mean (SD, range)	8.6 (7.0, 1 mth–24 yrs)	NT
	Females mean (SD, range)	8.9 (6.9, 1 mth–24 yrs)	NT
Duration of ART at time of MAM (months)	Males n = 45, mean (SD, range)	96.3 (77, 1–240)	NT
	Females n = 22, mean (SD, range)	67.9 (57.3, 1–192)	NT
Months suppressed (<400cpm) at time MAM	Males n = 42, mean (SD, range)	49.2 (46.7, 1–184)	NT
	Females n = 18, mean (SD, range)	49.8 (43.9, 1–149)	NT
Months suppressed (<40cpm) at time MAM	Males n = 31, mean (SD, range)	30 (37.8, 2–183)	NT
	Females n = 17, mean (SD, range)	30.1 (22.3, 1–66)	NT
ART naïve	Males (n)	12	NT
	Females (n)	2	NT

NT = Not tested.

**Figure 1:**
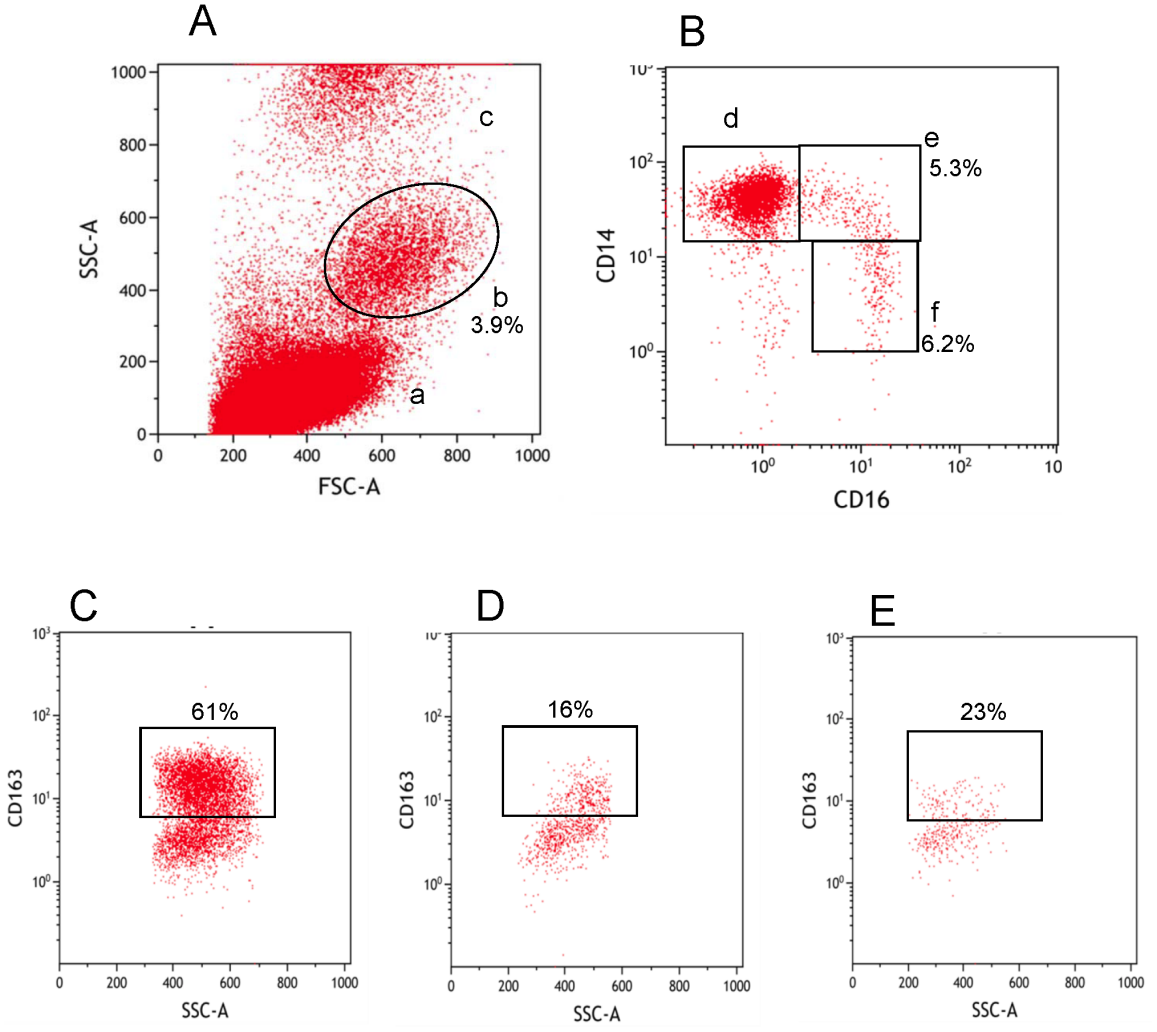
Phenotypic identification of monocytes and monocyte subsets. Whole blood monocytes were identified by a forward scatter and side scatter plot (A). Lymphocytes (a), monocytes (b) and the granulocytes (c) are shown. The three monocyte subsets were identified from the monocyte population (b) by CD14 and CD16 expression (B). Classical monocytes (d-CD14^++^/CD16^-^), intermediate monocytes (e-CD14^++^/CD16^+^) and non-classical monocytes (f-CD14^+^/CD16^+^) were gated for further analysis of activation markers. For example figures C, D and E show CD163 expression from d, e and f respectively.

The identification of monocyte subsets based on CD14 and CD16 expression was investigated in a whole blood flow cytometry assay as shown in Fig. 1. Upon identification of the monocyte subsets, the expression of CD64, CD143 and CD163 proteins were then assessed. For example, Fig. 1C, 1D and 1E show CD163 expression on classical, intermediate and non-classical monocytes respectively.

We initially investigated whether there was a difference in the proportion of monocyte subsets between HIV-infected and healthy control subjects. We found no influence of HIV status on either classical monocytes (HIV- median 78% (SE 1.4), HIV+ median 80% (SE 1.9), p = 0.75), intermediate monocytes (HIV median 4.2% (SE 0.5), HIV+ median 3.8% (SE 0.3), p = 0.18), or non classical monocytes (HIV median 5.3% (SE 0.3), HIV+ median 4.8% (SE 0.3), p = 0.65) using Mann-Whitney tests to compare groups. There was no detectable influence of gender (p>0.05, Mann-Whitney) or correlation with age (p>0.05, Spearman's rho) for any monocyte subset.

Comparing participants according to HIV viral load status (21 controls, 60 with ‘suppressed’ HIV RNA levels <400, and 21 with ‘viremic’ HIV RNA levels >400 copies/mL), we found significantly higher levels of intermediate CD14^++^/CD16^+^ monocytes in viremic HIV^+^ cases compared with suppressed HIV (p_adjusted_ = 0.01), although no significant differences between viremic HIV^+^ and control groups (p_adjusted_ = 0.95). This was associated with a corresponding trend on classical CD14^++^/CD16^-^ monocytes, in which levels were lower among viremic HIV^+^ patients compared with those with suppressed viral loads (p = 0.08). The proportion of non-classical CD14^+^/CD16^++^ monocytes was similar between all groups (p>0.45). There was no detectable association of CD4 T cell count or T cell CD4:8 ratio with any monocyte subset (all p≥0.7).

### Influence of HIV status on monocyte CD64, CD163 and CD143 cell surface expression

**Figure 2:**
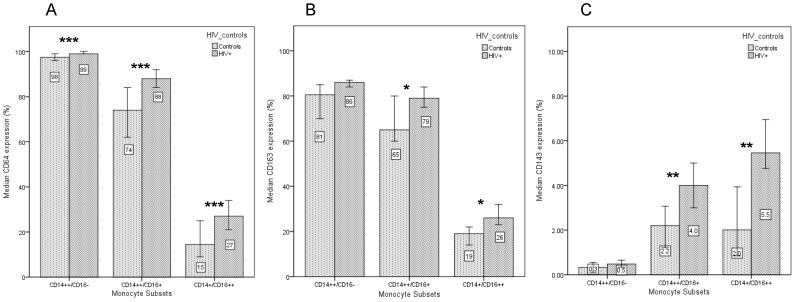
The median cell surface expression and 95% confidence intervals are shown for CD64 (A), CD163 (B) and CD143 (C) on monocyte subsets in HIV and control subjects. Expression on classical monocytes is significantly different between HIV and control groups for CD64 (p<0.001) but not for CD163 or CD143. Expression of all cell surface markers on CD16^+^ monocytes, however, are significantly different between the HIV infected and control groups (* p<0.05, ** p<0.01 *** p<0.001).

The expression of CD64, CD163 and CD143 proteins on all monocyte subsets was explored using whole blood immunophenotyping as described above. As expected and shown in Fig. 2, classical CD14^++^/CD16^-^ monocytes showed the highest overall levels of CD64 [1] and CD163 [10], [11] expression (98% and 79% respectively), while higher overall levels of CD143 expression were observed in CD16^+^ monocyte populations [9].

Comparing HIV infected and control groups (Mann-Whitney), we found that HIV status was associated with significantly higher CD64 expression on all three monocyte subpopulations (Fig. 2A) with a difference of approximately 14% in both intermediate CD14^++^/CD16^+^ monocytes (median (SE): 88% (1.2) vs 74% (3.3), p<0.001) and non-classical CD14^+^/CD16^++^ monocytes (27% (2.3) vs 14.5% (2.1), p<0.001).

HIV infection was also associated with higher monocyte CD163 expression for both intermediate CD14^++^/CD16^+^ monocytes (79% (2.2) vs 65% (3.5), p = 0.048) and non-classical CD14^+^/CD16^++^ monocytes (26% (1.9) vs 19% (2.1), p = 0.017) (Fig. 2B). Despite low overall CD143 expression, HIV status was associated with higher expression on CD14^++^/CD16^+^ intermediate monocytes (4.0% (0.6) vs 2.2% (0.3), p = 0.01) and CD14^+^/CD16^++^ monocytes (5.5% (0.9) vs 2.0% (0.4), p = 0.01) among HIV^+^ participants (Fig. 2C).

**Figure 3:**
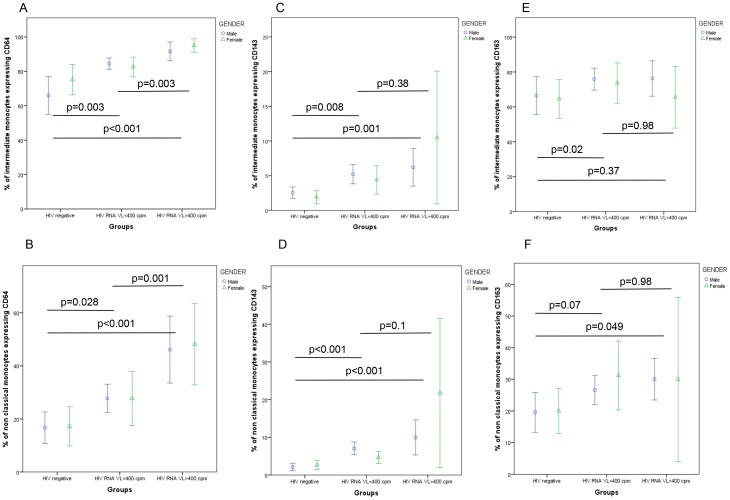
HIV viral load status has different influences on monocyte cell surface expression. Expression of CD64 on intermediate (A) and non-classical (B) monocytes, CD143 on intermediate (C) and non-classical (D) monocytes, and CD163 on intermediate (E) and non-classical (F) monocytes are shown below. (Data presented as mean values and 95% confidence intervals; p-values derived from Kruskal-Wallis tests).

We then sought to examine the association of plasma HIV RNA levels with these cell surface markers within the two CD16^+^ monocyte populations, comparing the normal healthy control group, HIV^+^ participants with viral suppression (viral load <400 copies/ml) and those with higher levels of viremia (HIV-1 viral load >400 copies/ml), with additional stratification by gender as shown in Fig. 3. This analysis revealed significant between-group differences in CD64 expression involving both intermediate monocytes (Fig. 3A, p<0.005) and non-classical monocytes (Fig. 3B, p<0.03), indicating that although effective ART was associated with reduced CD64 expression compared with viremic infection, levels were not normalised to those seen in the healthy control group. No additional influence of age or gender was detected in either of the CD16^+^ monocyte populations, in multivariate regression analyses (p>0.5). Similar multivariate analyses within the HIV^+^ group identified a marginal association of lower CD4 T cell counts as well as higher HIV RNA levels on CD64 expression in both intermediate monocytes (CD4 count, p = 0.08; HIV RNA, p = 0.004) and non-classical monocytes (p = 0.06, p = 0.001 respectively).

In contrast, we found that HIV^+^ status was associated with elevated CD143 expression levels on both CD16^+^ monocyte populations, regardless of viral load suppression (p<0.01 for all HIV^+^ vs. control comparisons, p>0.05 for comparisons of suppressed vs. viremic HIV^+^ groups), although overall effect sizes were small as shown in Fig. 3C and 3D. Similarly, higher levels of CD163 expression on intermediate CD14^++^/CD16^+^ monocytes were observed in the suppressed HIV^+^ group compared to controls (Fig. 3E; p = 0.02) whilst in the non-classical CD14^+^/CD16^++^ monocytes higher levels were observed in the viremic HIV^+^ group compared to controls (Fig. 3F; p = 0.049), with no discernible difference between the suppressed and viremic groups (p = 0.98). No significant influence of age, gender or CD4 T cell count on CD143 or CD163 monocyte expression in any monocyte population could be detected in regression analyses, including within analyses restricted to the HIV+ study group (all p>0.2).

### Influence of HIV status on plasma levels of sCD14, sCD163 and CXCL10

**Figure 4:**
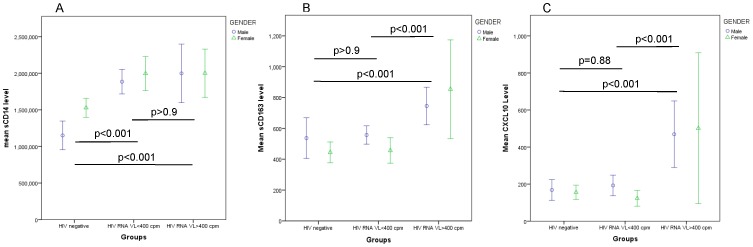
sCD14 levels are significantly elevated in HIV patients irrespective of HIV viral load status (A). In contrast sCD163 levels are only elevated in viremic HIV patients (B) and a similar relationship is seen with CXCL10 levels (C). (Data presented as mean values and 95% confidence intervals; p-values are derived from log transformed data and ANOVA tests were adjusted for multiple comparisons).

Comparing normalised (log-transformed) levels of plasma biomarkers in the HIV-infected and healthy control groups (ANOVA with Bonferroni correction as appropriate for multiple comparisons), we found a highly significant difference in plasma sCD14 levels in the HIV^+^ group compared to controls (Fig. 4A; p<0.001), with no detectable influence of HIV suppression on sCD14 levels compared with the viremic HIV group (p>0.9). Age and gender also had no influence in adjusted analyses (p = 0.72 and p = 0.21 respectively). Restricting analyses to the HIV^+^ group, there was no evidence of an association between sCD14 and plasma HIV RNA levels (p = 0.7) or CD4 T cell count or T cell CD4:8 ratio (both p>0.6).

Plasma levels of sCD163 were also elevated in the HIV group overall (p<0.05), but in striking contrast to sCD14, we found that sCD163 was significantly increased in viremic compared to both virally-suppressed patients (fig. 4B; p<0.001) and healthy controls (p<0.001) whilst sCD163 levels in suppressed patients were similar to healthy controls (p>0.9). Within the HIV^+^ group, sCD163 levels were independently associated with plasma HIV RNA levels (p<0.01) as well as T cell CD4:8 ratio (p = 0.003) in multivariate analysis, with no additional effect of age (p = 0.3) or gender (p = 0.7).

Elevated CXCL10 levels were also specifically associated with viremic HIV infection compared to both controls (Fig. 4C:p<0.001) and suppressed HIV groups (p<0.001), with no additional effect of gender (p = 0.3) or age (p = 0.8). CXCL10 levels were associated with plasma HIV RNA levels (p<0.001) but not absolute CD4 T cell count (p = 0.1) or CD4:8 ratio (p = 0.5) within the HIV+ group.

We also investigated if the duration of effective HIV treatment had an influence on these plasma biomarker levels, considering 48 patients who had achieved virological suppression <40 copies/mL for at least one month prior to assessment (31 males and 17 females, with mean duration of viral suppression 30 months (SD 6.8) and 30 months (SD 5.4) respectively). Here, the duration of virological suppression had no detectable influence on plasma levels of CXCL10 (p = 0.47), sCD163 (p = 0.17) or sCD14 (p = 0.49). Similar results were obtained when considering viral load levels <400 copies/mL as evidence of virological suppression (42 males and 18 females, mean duration of viral suppression <400 copies/mL 49 months (SD 7.2) and 50 months (SD 10.3), with no evidence of an association with CXCL10 (p = 0.29) or sCD14 (p = 0.65). There was a trend observed for sCD163 (p = 0.08), although this lost significance (p = 0.34) after adjustment for the influence of CD4:8 ratio (p = 0.008) noted earlier. No significant effect of HIV treatment duration, or time since HIV diagnosis, was observed for plasma biomarker levels (all p>0.3).

### Correlations between flow cytometry and plasma biomarkers

Comparing plasma biomarkers in the first instance, we found that CXCL10 levels strongly correlated with sCD163 levels in the overall dataset (r = 0.50, p<0.001) and within the HIV+ group (r = 0.58, p<0.001), while there was no correlation between plasma levels of sCD14 and CXCL10 (r = 0.08, p = 0.5) or sCD163 (r = 0.06, p = 0.5).

We next sought to examine correlations between plasma biomarkers and flow cytometry expression profiles within this study population, with investigation of significant bivariate correlations in subsequent multivariate regression analysis with log-transformed plasma biomarkers as the dependent variable. Here we found that sCD14 correlated independently with non-classical monocyte CD143 expression (r = 0.3, p = 0.006), as well as with intermediate monocyte expression levels of CD64 (r = 0.3, p = 0.001) providing a modest overall R^2^ value of 0.07 (p = 0.03). The significance of these variables was lost after inclusion of HIV status as a covariate (β 0.34, p = 0.003), suggesting that HIV infection *per se* was the dominant determinant of sCD14 levels. For CXCL10, we found that a combination of sCD163 (β 0.43, p<0.001) and non-classical monocyte CD64 expression (β 0.30, p = 0.001) provided the best model (R^2^ 0.37, p<0.001), with no evidence of an additional effect of HIV status (p = 0.22). Soluble CD163 variability was best explained by a model including CXCL10 (β 0.47, p<0.001), although in this case combined with non-classical monocyte CD143 expression (β 0.27, p = 0.002). This final model (R^2^ 0.36, p<0.001) was also unaffected by the addition of HIV status (p = 0.54). No effect of age, gender or CD4 count was detected.

## Discussion

This study was primarily concerned with the evaluation of methods relevant to monocyte activation during HIV infection and treatment, which could be readily adopted in routine laboratory practice. This may be particularly relevant in the current treatment era, when the availability of effective HIV therapy (defined by suppression of plasma HIV RNA levels and recovery of normal CD4^+^ T cell counts) has brought into question the prognostic value of regular CD4 T cell monitoring once these treatment goals have been achieved [20]. In this context, there is an opportunity to explore novel aspects of chronic HIV infection that currently remain ‘invisible’ in routine care despite being prognostically important [14], [15], and which may not be influenced by what would otherwise be considered effective therapy.

The results of our flow cytometric analyses strongly support the concept that monocytes are phenotypically altered in the presence of HIV infection, with a skewing of the distribution towards CD16-expressing monocytes that have been previously associated with a range of adverse outcomes in both HIV-infected and non-infected populations [7]–[9]. We also observed increased cell surface expression of CD64, CD143 and CD163 on these CD16^+^ monocytes, which were not reduced to healthy control levels despite virologically-suppressive ART. These results suggest that chronic HIV infection promotes CD16^+^ monocyte activation, overriding the ‘default’ apoptotic program that generally limits monocyte maturation and monocyte survival (with a half-life of approximately three days) [21]. Within this highly dynamic system, the persistence of pro-inflammatory cell surface markers on CD16^+^ monocytes in the face of suppressive HIV treatment is noteworthy.

Measurement of the plasma biomarkers sCD14, sCD163 and CXCL10 in this cohort also revealed distinct associations with HIV disease and treatment. In these analyses, sCD14 levels were significantly elevated in the HIV^+^ group, irrespective of viral load level, CD4 T cell counts, or demographic variables. This stability of sCD14 levels has been previously noted over extended periods of follow-up among patients on HIV therapy [14], [19], [22], with further evidence that sCD14 levels are established at a ‘set point’ level very early in the course of HIV infection [23]. The fact that sCD14 levels are chronically increased with HIV infection compared to controls in this and other studies [22], [24], and that sCD14 levels are also strongly associated with mortality risk in the general population [25] as well among those with HIV infection [14], suggest that variability is prognostically important and is determined by HIV-specific factors as well as those shared among the general population. In this respect, a recent genome wide association study has identified heritable factors accounting for ∼30% of this variation, while sCD14 levels were also significantly associated with cardiovascular risk factors (smoking, diabetes mellitus, fasting glucose and hypertension; all p<0.001) as well as with C-reactive protein and IL-6 [25]. Similar associations between sCD14 and cardiovascular risk factors have also been discovered in a study of HIV-infected patients [26].

In contrast, we found that both CXCL10 and sCD163 levels were elevated in association with viremic HIV infection – with significant linear correlations between plasma HIV RNA levels and these variables – but that suppressive ART restored plasma levels to healthy control values. In the case of CXCL10 this is in keeping with previous studies [18], [19], as well as observations in acute HIV-1 infection that plasma CXCL10 levels provide a very early predictive measure of set-point viral load, CD4 T cell activation and disease progression [27] most likely through direct stimulation of monocyte and plasmacytoid dendritic cell CXCL10 production via toll-like receptor ligation [18]. Similar evidence has been provided for a correlation between plasma HIV RNA levels and sCD163 [17], and the interrelationship between sCD163 and CXCL10 identified in this study has also been noted in rheumatoid arthritis [28] – suggesting common activation pathways for these molecules.

In terms of the utility of these assays in a routine HIV laboratory setting, our data indicate that virological suppression (a known variable in HIV care) would be anticipated to have a favourable influence on plasma CXCL10 and sCD163 levels – potentially limiting the independent value of these tests in routine management. In contrast, many of the observed effects of HIV infection on CD16^+^ monocyte surface expression profiles (CD64, CD143 and CD163) and plasma sCD14 levels were not corrected by HIV treatment, nor were they associated with other variables considered in these analyses (gender, age, CD4 T cell count). Given available evidence that these monocyte biomarkers have prognostic significance in the general population as well as among those with HIV infection – including when standard CD4 T cell monitoring approaches carry limited prognostic value in the setting of ‘successful’ therapy [20], [29] – we would suggest that consideration of their inclusion in routine care is warranted. This is particularly relevant in light of emerging HIV treatment strategies that seek to reduce systemic immune activation (including sCD14 levels), either through modifying antiretroviral therapy [30], [31], or introducing adjunctive therapies such as statins [32], low-dose corticosteroids [33], or IL-7 treatment [34]. In these circumstances the ability to assess who will benefit from these treatment strategies, and then to monitor treatment response, will be critically important.
